# Evidence of *Bovine viral diarrhea virus* Infection in Three Species of Sympatric Wild Ungulates in Nevada: Life History Strategies May Maintain Endemic Infections in Wild Populations

**DOI:** 10.3389/fmicb.2016.00292

**Published:** 2016-03-09

**Authors:** Peregrine L. Wolff, Cody Schroeder, Caleb McAdoo, Mike Cox, Danielle D. Nelson, James F. Evermann, Julia F. Ridpath

**Affiliations:** ^1^Nevada Department of Wildlife, RenoNV, USA; ^2^Nevada Department of Wildlife, ElkoNV, USA; ^3^Veterinary Microbiology and Pathology, College of Veterinary Medicine, Washington State University, PullmanWA, USA; ^4^Veterinary Clinical Medicine and Washington Animal Disease Diagnostic Laboratory, College of Veterinary Medicine, Washington State University, PullmanWA, USA; ^5^Ruminant Diseases and Immunology Research Unit, National Animal Disease Center, United States Department of Agriculture – Agricultural Research Service, AmesIA, USA

**Keywords:** *bovine viral diarrhea virus*, bighorn sheep, mountain goat, mule deer, Nevada, *Odocoileus hemionus*, *Oreamnos americanum*, *Ovis canadensis*

## Abstract

Evidence for *bovine viral diarrhea virus* (BVDV) infection was detected in 2009–2010 while investigating a pneumonia die-off in Rocky Mountain bighorn sheep (*Ovis canadensis, canadensis)*, and sympatric mountain goats (*Oreamnos americanum*) in adjacent mountain ranges in Elko County, Nevada. Seroprevalence to BVDV-1 was 81% (*N* = 32) in the bighorns and 100% (*N* = 3) in the mountain goats. Serosurveillance from 2011 to 2015 of surviving bighorns and mountain goats as well as sympatric mule deer (*Odocoileus hemionus*), indicated a prevalence of 72% (*N* = 45), 45% (*N* = 51), and 51% (*N* = 342) respectively. All species had antibody titers to BVDV1 and BVDV2. BVDV1 was isolated in cell culture from three bighorn sheep and a mountain goat kid. BVDV2 was isolated from two mule deer. Six deer (*N* = 96) sampled in 2013 were positive for BVDV by antigen-capture ELISA on a single ear notch. Wild ungulates and cattle concurrently graze public and private lands in these two mountain ranges, thus providing potential for interspecies viral transmission. Like cattle, mule deer, mountain goats, and bighorn sheep can be infected with BVDV and can develop clinical disease including immunosuppression. Winter migration patterns that increase densities and species interaction during the first and second trimester of gestation may contribute to the long term maintenance of the virus in these wild ungulates. More studies are needed to determine the population level impacts of BVDV infection on these three species.

## Introduction

The pestivirus *bovine viral diarrhea virus* (BVDV) is considered an important disease of cattle, and infection also occurs in other domestic and wild ruminants (Passler and Walz, 2010). BVDV infection has been documented through serosurveillance and virus isolation in a number of captive and free ranging North American ungulate species including, Rocky Mountain bighorn sheep (*Ovis canadensis, canadensis*; Van Campen et al., 2003) mountain goats (*Oreamnos americanum*; Nelson et al., 2008), white-tail deer (*Odocoileus virginianus*; Pogranichniy et al., 2008; Wolf et al., 2008; Kirchgessner et al., 2013) mule deer (*Odocoileus hemionus*; Van Campen et al., 2001; Roug et al., 2012), elk (*Cervus elaphus*; Tessaro et al., 1999), moose (*Alces alces*; Kocan et al., 1986), bison (*Bison bison*; Taylor et al., 1997) pronghorn (*Antilocapra americanum*; Dubay et al., 2006) and caribou (*Rangifer tarandus*; Morton et al., 1990).

*Bovine viral diarrhea virus* can cause clinical disease including gastrointestinal and respiratory disease, reproductive loss, and lymphoid depletion causing immunosuppression in susceptible ungulates. Infection of pregnant females during the first trimester of pregnancy may also produce persistently infected (PI) young. Immunotolerant to the virus, PI animals are life-long and efficient shedders and are the primary transmitters of virus to cohorts, although transiently infected (TI) animals may also play a significant role in virus transmission (Thurmond, 2005). PI individuals have been reported in free-ranging mule (Duncan et al., 2008) and white-tail deer (Chase et al., 2008) and in captive mountain goats (Nelson et al., 2008). PI white-tail deer fawns were produced when dams were exposed to PI cattle (Passler et al., 2009) and white-tail deer (Passler et al., 2010), or experimentally infected (Passler et al., 2007; Ridpath et al., 2008) during the first trimester of gestation. Although contact with domestic cattle is considered the likely source of introduction of BVDV into free-ranging ruminant populations (Kocan et al., 1986; Nielsen et al., 2000), the virus can be maintained and is likely endemic in some North American wildlife populations.

We identified BVDV infection over time in sympatric Rocky Mountain bighorn sheep, mountain goats, and mule deer on adjacent mountain ranges [East Humboldt range (EHR) and Ruby Mountains (RMs)] in Elko County, Nevada. We propose that the virus has become endemic within all three mountain ungulate species. The timing of movement to and residence on winter range, which occurs during the first two trimesters of pregnancy in all species, results in increased animal densities and species overlap. Increased contact between and within species could potentiate transmission and perpetuate virus maintenance within these populations. Impacts of BVDV infection on population health and annual recruitment could not be quantified in this study but bears further investigation.

## Animal Handling

All capture, handling and disease surveillance activities were approved and conducted under the direction of the Nevada Department of Wildlife (NDOW). Live animal sampling was conducted in January and February following helicopter net gun capture. In addition 20 bighorn sheep were captured via ground darting (Pneu-dart, Williamsport, PA, USA) using BAM^TM^ (Wildlife Pharmaceuticals, Inc., Windsor, CO, USA) as previously described (Wolfe and Miller, 2009). Blood samples were collected using routine jugular venipuncture. Ear notch samples were taken in a standard manner utilizing a v-cut ear notcher, producing a base cut of 8 mm with 10 mm to tip (Nasco, Salida, CA, USA).

## Laboratory Analysis

Serum virus neutralization (SN) for BVDV1 antibody titers was conducted at the Washington Animal Disease Diagnostic Laboratory, College of Veterinary Medicine, Pullman, Washington (WADDL) utilizing Singer strain as previously described (World Organization for Animal Health, 2008) and BVDV2 utilizing strain 125 at Oregon State University, Veterinary Diagnostic Laboratory in Corvallis, Oregon (OSU-VDL) as previously described by (Montrose et al., 2015). All histopathology, immunohistochemistry for pestivirus and PCR for BVDV on fresh and archived tissues blocks was performed at WADDL as previously described (Nelson et al., 2008). Virus isolation was conducted on fresh tissue and whole blood and pestivirus typing by PCR on serum at USDA–ARS as previously described by (Ridpath et al., 2008). Ear notches submitted to USDA-ARS were screened for BVDV using the antigen-capture ELISA (ACE; Herdchek^®^, Idexx Laboratories, Westbrook, ME, USA) as previously described (Ridpath et al., 2008).

## BVDV in Rocky Mountain Bighorn Sheep

Rocky Mountain bighorn sheep were introduced into the RM in 1989–1990 and the EHR in 1992. In the winter of 2009–2010, approximately 91% (population estimate 175) and 95% (population estimate 140) of the herds respectively, were lost due to an all age bacterial pneumonia die-off. During the disease investigation VN titers were detected to BVDV1 in 81% (*N* = 32) of the sheep with 77% having titers ≥ 1:512. Fifteen also had a seroprevalence for BVDV2 of 93% (titers ranged from 1:8 to 1:256). Histologic lesions from mortalities recovered during the pneumonia die-off were consistent with bacterial pneumonia and non-specific for pestivirus disease. Archived samples from the 2009 to 2010 die-off were submitted to USDA-ARS including serum from six animals for PCR and tissues from three mortalities for virus isolation. Four of the six sera were positive by PCR for BVDV2. Two of these animals were seronegative to BVDV1 at the time of capture but no serum was submitted for BVD2 and the others had VN antibody titer to BVDV1 of 1:128 and 1:512 and to BVDV2 of 1:16 and 1:64 respectively. BVDV1 was isolated from tissue in these three mortalities. Only one of these animals, an adult ram, was sampled prior to death and was seronegative for BVDV1 and no serum was submitted for VN for BVDV2. Paraffin-embedded splenic tissue from this ram was negative for BVDV by PCR but positive for pestivirus on immunohistochemistry.

**FIGURE 1:**
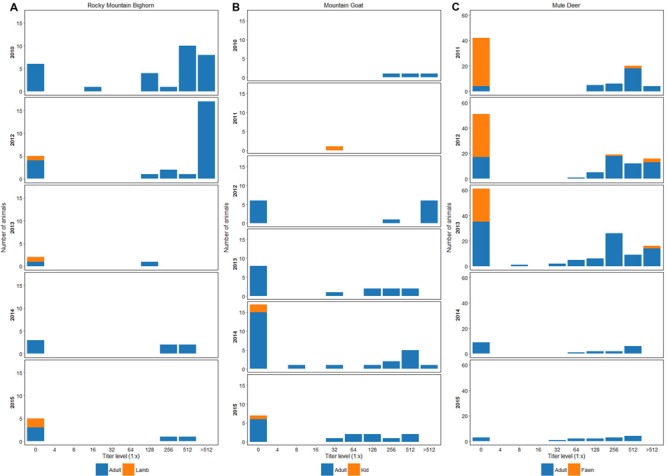
**(A)** Seroprevalence and antibody titers to BVDV1 in bighorn sheep sampled in the RM and EHR from 2010 to 2015. **(B)** Seroprevalence and antibody titers to BVDV1 in mountain goats sampled in the EHR and RM from 2010 to 2015. **(C)** Seroprevalence and antibody titers to BVDV1 in mule deer from 2010 to 2015. In years where fawns were sampled, 1–2 each year have had virus neutralization titers of 1:256 or greater to BVDV-1. Fawns were 7–8 months-old at capture and titers could represent maternal antibody or naturally acquired infection.

Subsets of the survivors and their offspring have been sampled annually since 2012. Seroprevalence to BVDV1 was 80% (*N* = 26) in 2012, 33% (*N* = 3) in 2013, 57% (*N* = 7) in 2014, and 28% (*N* = 7) in 2015 (**Figure 1A**). In 2013 and 2014 a total of 10 non-paired ear notch samples were tested by ACE. All samples were negative.

## BVDV in Mountain Goats

Mountain goats were introduced into the RM in 1964 and ‘67 and into the EHR in 1981. In the winter of 2009–2010 approximately 30% (population estimate 220) and 13% (population estimate 130) of the herds respectively, were lost to an all age bacterial pneumonia die-off. During the disease investigation three mountain goats in the EHR were sampled and all were seropositive to BVDV1, with two having BVDV1 titers of ≥1:512, and all having titers to BVDV2 of 1:32. In 2011 an approximately 8 weeks-old, male kid was found in the RM. The animal was surrendered to the NDOW but died 36 h later. Histopathology revealed a bacterial bronchopneumonia, and necrotizing mesenteric lymphadenitis suggested the possibility of BVDV infection. Though immunohistochemistry on paraffin embedded tissue blocks of intestine, lung and lymph node was negative, virus isolation on archived splenic tissue was positive for BVDV1. The VN titer for BVDV1 was 1:32; no serum was submitted for VN to BVDV2.

Subsets of die-off survivors and their offspring have been captured annually since 2012 for marking and disease surveillance. Seroprevalence by VN to BVDV1 in 2012 was 88% (*N* = 9), 46% (*N* = 15) in 2013, 41% (*N* = 24) in 2014, and 60% (*N* = 15) in 2015 (**Figure 1B**). In 2013 and 2014 a total of 30 non-paired ear notch samples were tested by ACE; all samples were negative. In 2015, three mountain goat kid mortalities were recovered from the EHR. Two died from bacterial bronchopneumonia at 8–10 weeks of age and one was a perinatal death. Splenic tissues (*N* = 2) and lung (*N* = 1) were tested for BVDV by PCR and were negative. Histopathology lesions were non-specific for BVDV in all three kids.

## BVDV in Mule Deer

Mule deer are native to Nevada. The population of deer in the management units which include the RM and EHR from 2011 to 2015 was estimated at 20,000 (NDOW, unpublished data). A migration study conducted between 2011 and 2015 involved the capture and sampling of 342 deer (236 adults and 106 fawns). All deer received radio collars which emit a mortality signal if no movement is detected from the animal for 8 h and all mortalities were investigated; however, none were recovered within a timeframe to determine if infectious disease was the cause of death.

Seroprevalence to BVDV1 across all age classes was 35% (*N* = 101) in 2011; 52% (*N* = 108) in 2012; 48% (*N* = 117) in 2013; 55% (*N* = 20) in 2014; and 80% (*N* = 15) in 2015 (**Figure 1C**). Seroprevalence to BVDV1 for fawns (estimated age 7 and 8 mos.) across all sample years was 7.5% (*N* = 106) with 62% (*N* = 8) having titers of ≥1:512. Serum samples from 33 deer with BVDV1 titers ≥ than 1:512 were submitted for endpoint titers as well as VN titers for BVDV2. Twenty four percent of deer had endpoint titers of 1:2048 and 12% had endpoint titers of 1:1024 to BVDV1 and from 1:32 to 1:1024 for BVDV2.

In 2013, 84 non-paired ear notch samples were tested by ACE. Six animals were positive, two adult females and four fawns. One fawn had a titer to BVDV1 of ≥1:512 the rest of the deer were seronegative. BVDV2 was isolated from the ear notch of another fawn. Three of four fawns died 3–4 months after capture of trauma, predation, or unknown cause. One fawn was confirmed alive at 11 months post-capture but dropped his collar and was lost to follow up. One adult female died of trauma and one was presumed predated 7 and 17 mos. post-capture respectively. BVDV2 was isolated from whole blood from another doe whose single ear notch sample was ACE negative. This doe was VN negative for BVDV1 and BVDV2. In 2014, 19 non-paired ear notch samples collected from adult does were tested by ACE; all were negative.

## Discussion

Infection with BVDV1 and BVDV2 was detected over a 5 year period based on serology, serum and tissue PCR, antigen-capture ELISA, and virus isolation from three wild ungulate species in adjacent mountain ranges in northeastern Nevada. These findings are consistent with endemic infection in these sympatric populations.

Previous studies have found a high prevalence of VN titers in cervid populations (Van Campen et al., 2001; Lillehaug et al., 2003; Passler et al., 2008) and in other wild ungulates (Passler and Walz, 2010) suggests endemic infection. In this study, repeated serosurveillance from 2010 to 2015 indicated a high percentage of VN titers to BVDV1 in the bighorn sheep (28–80%), mountain goats (25–50%), and mule deer (35–80%) and to BVDV2 in tested animals: bighorn sheep 93% (*N* = 15), mountain goats 100% (*N* = 3), and mule deer 100% (*N* = 33). Seroprevalence to BVDV1 in lambs was 0% (*N* = 4), kids 25% (*N* = 4), and fawns 7.5% (*N* = 106). It is unknown when maternal antibodies to BVDV wane, so positive titers may represent maternally derived antibodies or postnatally acquired infection, but either scenario suggests that BVDV1 and BVDV2 are circulating in these populations.

BVDV1 and BVDV2 were detected by virus isolation, PCR, or ACE in all three species. BVDV1 was isolated from bighorn sheep and a mountain goat kid. Two of four sheep that were PCR positive for BVDV2 were seronegative for BVDV1 with no serum submitted for BVDV2, and the other PCR-positive sheep were seropositive for both. Cross reaction can occur in serologic assays between strain antibodies which could account for the titers to BVDV1 in these sheep. Alternatively, the BVDV1 titers were due to a previous transient infection. BVDV2 was also isolated from a doe and a fawn. The doe was ACE negative and the fawn ACE positive; both were VN seronegative for BVDV1 and BVDV2. The ACE is considered to be 90–95% specific for identifying PI in cattle, but is less sensitive for detection of acute or transient infection; and ACE has not been validated in wild ruminants. Most likely the fawn was PI, and the doe may have been acutely infected or PI with BVDV2. Five other mule deer were also positive by ACE. One fawn had a BVDV1 titer of ≥1:512 which may indicate that he was PI with BVDV2 and was either acutely infected with, or had maternal antibodies to, BVDV1. Without repeated tests, it is not possible to definitively determine whether these individuals that had direct evidence of BVDV infection are TI or PI. However, our data suggests both conditions exist in these sympatric populations providing the means for maintaining the virus over time.

**FIGURE 2:**
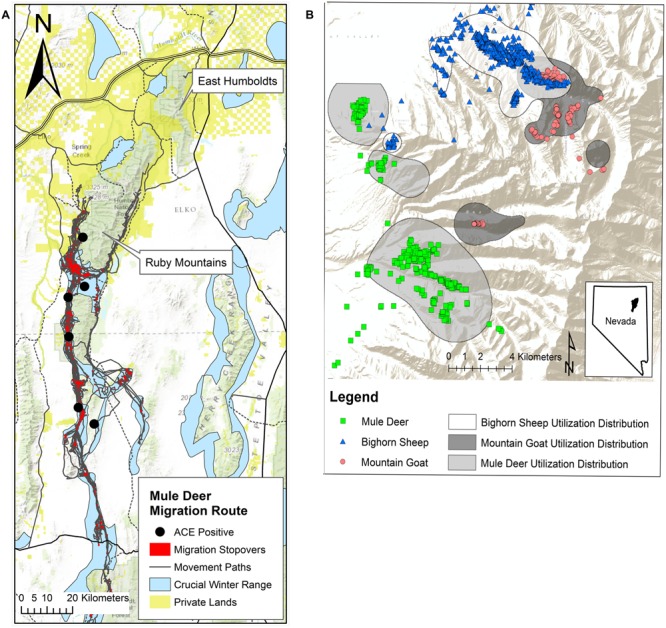
**(A)** Population-level migration route (gray) and stopover sites (red) estimated for the Ruby Mountain (RM) mule deer herd, 2012–2013 (Sawyer and Brittell, 2014) population estimate of 20,000. Approximately 80% of this herd exhibits a long distance migratory strategy to winter range (blue) while the remaining 20% are considered resident deer exhibiting only altitudinal movement. Autumn migration on average begins around October 15 and can last for up to 90 days. In spring deer begin to migrate back from winter range starting mid-March to mid-April. Migrating deer may spend 90% of their time at these stopover sites along the migration route and GPS collar data (12 locations/day for 18 months) for two mule deer indicated that individuals can remain in these stopover areas for 1–2 months. This migration pattern increases densities during the first 2 months of gestation increasing the risk that naïve does could be exposed to BVDV. Gold squares represent private land holdings, primarily cattle ranches that run along the base of the mountain providing an opportunity for interspecies contact. Black circles indicate capture locations of the six deer that were antigen-capture ELISA positive on a single ear notch sample taken in 2013. **(B)** Global positioning system (GPS) multiple daily location collar data from December 1, 2012 to February 15, 2013, for mountain goats (887 location points, *N* = 5) and bighorn sheep (2622 location points, *N* = 7) in the northern portion of the RMs. Blue triangles and red circles illustrate winter range overlap between the two species. Population densities are increased during the first trimester of pregnancy when animals move to and congregate on winter range thus increasing the risk that naïve ewes and nannies could be exposed to BVDV. There is also limited overlap (green squares/blue triangles) occurring between bighorn sheep and mule deer (1480 location points, *N* = 8).

The history of BVDV infection within susceptible domestic and wild ungulate populations in this region has not been documented; thus a domestic origin of the virus has not been definitively determined. The EHR and RM encompass 3 billion hectares, and are comprised of public and private land, and Federal livestock grazing allotments cover 60–70 and 95% of these mountain ranges, respectively. Private ranches surround both mountains and are the primary source of the cattle which graze the allotments (**Figure 2A**). Radio collar data and visual observations from aerial and ground surveys confirm that temporal and spatial overlap occurs between wild ungulates and domestic cattle and between these wildlife species. Although domestic cattle have grazed these mountains for decades, infection with BVDV was first detected in 2010 in bighorn sheep and mountain goats and 2011 in mule deer. Prior serosurveillance for BVDV was not conducted, so it is unknown when spill-over from cattle to wildlife or transmission between wildlife species may have occurred.

Seasonal migration and reproductive timing likely play important roles in transmission and potential maintenance of the virus in these ungulate populations. Seasonal migration results in congregation of these animals on winter range increasing animal densities and the chance for intra- or interspecies viral transmission (Van Campen et al., 2001; Wolf et al., 2008; Passler et al., 2010). After rut, which peaks in mid to late November for all three species, global positioning system (GPS) collar data confirmed that each species moves to, or are on, their respective winter range through the first trimester of gestation: 0–60 days in bighorn sheep (Lawson and Johnson, 1987) and mountain goats (Wigal and Coggins, 1987) and 0–66 days in mule deer (Mackie et al., 1987). Infection of a naïve dam during gestation can produce PI offspring, as experimentally proven in white-tail deer (Passler et al., 2010). Thus increased densities along migration routes, converging winter ranges and reproductive timing in these species likely provides an ideal environment for virus transmission and maintenance in a population (**Figures 2A,B**).

The importance of BVDV infection with regard to morbidity and mortality is not known in these populations. Whether BVDV infection in the bighorn sheep or mountain goats played a significant role in the 2009–2010 pneumonia die-offs is also not clear. The 2009–2010 disease event in the EHR and the RM was attributed to pneumonia caused by *Mycoplasma ovipneumoniae* and secondary infection with *Pasteurellaceae* sp. (Besser et al., 2012; Shanthalingam et al., 2014) consistent with the majority of all-age, die-offs that have been reported in bighorn sheep in the western United States since 1980 (Cox and Carlsen, 2012). Possibly immunosuppression resulting from BVDV infection may have been a predisposing factor for the 2009–2010 pneumonia events. Two free-ranging bighorn sheep (Van Campen et al., 2003) as well as two captive mountain goats (Nelson et al., 2008) that presented with bacterial pneumonia had concurrent BVDV infection. In contrast, pneumonia die-offs were documented in two additional bighorn sheep herds in Nevada in 2011, with no serologic evidence of BVDV infection (NDOW, unpublished data). Experimental infection of adult and young mule deer (Van Campen et al., 1997), white-tail deer (Ridpath et al., 2008, 2012; Raizman et al., 2009; Passler et al., 2010) and elk (Tessaro et al., 1999) with BVDV caused subclinical to severe clinical disease and immunosuppression. Although, mule deer mortalities were investigated; infectious disease could not be confirmed as contributing to the death. We have not directly associated clinical disease with BVDV infection in this study, However, our findings strongly support that further testing for BVDV, should be included when investigating cases of respiratory disease in at risk wildlife species.

The importance of BVDV-induced reproductive disease in these wild ungulate species is unknown. Reproductive loss including fetal resorption, fetal mummification, abortion, weak fawns, and PI fawns has been documented in white-tail deer infected during the first and second trimesters of gestation (Ridpath et al., 2008, 2012; Passler et al., 2009, 2010), however, experimental infection of white-tail does during the third trimester of gestation did not affect reproduction (Ridpath et al., 2012). Autumn aerial surveys of mule deer between 1998 and 2008 indicated a significant drop in fawn recruitment which could not be fully explained by typical population drivers such carrying capacity, climatic conditions or predation. Recent surveys indicate fawn recruitment has slightly increased (NDOW unpublished data), but bighorn and mountain goat populations have not recovered after the 2009–2010 die-off. On-going annual losses of lambs at approximately 4–12 weeks of age from bacterial pneumonia is an epidemiologic feature in some bighorn sheep die-offs associated with mixed infections of *M. ovipneumoniae* and *Pasteurellaceae* sp. (Cox and Carlsen, 2012; Cassirer et al., 2013). In the EHR we noted a similar pattern in mountain goats with kids developing clinical signs and dying of bacterial pneumonia between 8 and 12 weeks of age (NDOW unpublished data) with no evidence of active BVDV infection. Further investigation to determine if infection with BVDV is affecting fawn, lamb and kid production, early survival or recruitment in these populations is warranted.

## Conclusion

Wild ungulates and cattle concurrently graze public and private lands in the EHR and RM creating potential for interspecies BVDV transmission. Ideal conditions for viral transmission such as the production of PI animals from pregnant naïve animals or a virulent strain spillover (Thurmond, 2005; Evermann, 2006) likely occurred perpetuating infection in three previously naïve species. Winter range overlap between bighorn sheep and mountain goats and philopatric mule deer migration patterns and timing may have further contributed to the transmission and potential maintenance of the virus in these populations. The impacts of BVDV infections on the health and recruitment in these three species are unknown creating an unpredictable variable confounding management of wild ungulate populations in Nevada.

## Author Contributions

Substantial contributions to the conception or design of the work; or the acquisition, analysis, or interpretation of data for the work: PW, CS, CM, MC, JR, JE, DN. Drafting the work or revising it critically for important intellectual content PW, CS, CM, MC, JR, JE, DN. Final approval of the version to be published: PW, CS, CM, MC, JR, JE, DN. Agreement to be accountable for all aspects of the work PW, CS, CM, MC, JR, JE, DN.

## Conflict of Interest Statement

The authors declare that the research was conducted in the absence of any commercial or financial relationships that could be construed as a potential conflict of interest.
